# Lifestyle Risk Factors for Weight Gain in Children with and without Asthma

**DOI:** 10.3390/children4030015

**Published:** 2017-02-25

**Authors:** Megan E. Jensen, Peter G. Gibson, Clare E. Collins, Jodi M. Hilton, Lisa G. Wood

**Affiliations:** 1 Priority Research Centre Grow Up Well, School of Biomedical Science & Pharmacy and School of Medicine & Public Health, Faculty of Health & Medicine, University of Newcastle, Newcastle, NSW 2308, Australia; 2 Priority Research Centre Healthy Lungs, School of Medicine & Public Health, Faculty of Health & Medicine, University of Newcastle, Newcastle, NSW 2308, Australia; peter.gibson@hnehealth.nsw.gov.au; 3 Department of Respiratory & Sleep Medicine, John Hunter Hospital, Newcastle, NSW 2310, Australia; 4 Priority Research Centre in Physical Activity & Nutrition, School of Health Sciences, Faculty of Health & Medicine, University of Newcastle, Newcastle, NSW 2308, Australia; clare.collins@newcastle.edu.au; 5 Paediatric Respiratory & Sleep Medicine, John Hunter Children’s Hospital, Newcastle, NSW 2310, Australia; jodi.hilton@hnehealth.nsw.gov.au; 6 Priority Research Centre Grow Up Well and Priority Research Centre Healthy Lungs, School of Biomedical Science & Pharmacy, Faculty of Health & Medicine, University of Newcastle, Newcastle, NSW 2308, Australia; lisa.wood@newcastle.edu.au

**Keywords:** diet quality, asthma, sleep, nutrition, paediatric, weight, obesity

## Abstract

A higher proportion of children with asthma are overweight and obese compared to children without asthma; however, it is unknown whether asthmatic children are at increased risk of weight gain due to modifiable lifestyle factors. Thus, the aim of this cross-sectional study was to compare weight-gain risk factors (sleep, appetite, diet, activity) in an opportunistic sample of children with and without asthma. Non-obese children with (*n* = 17; age 10.7 (2.4) years) and without asthma (*n* = 17; age 10.8 (2.3) years), referred for overnight polysomnography, underwent measurement of lung function, plasma appetite hormones, dietary intake and food cravings, activity, and daytime sleepiness. Sleep latency (56.6 (25.5) vs. 40.9 (16.9) min, *p* = 0.042) and plasma triglycerides (1.0 (0.8, 1.2) vs. 0.7 (0.7, 0.8) mmol/L, *p* = 0.013) were significantly greater in asthmatic versus non-asthmatic children. No group difference was observed in appetite hormones, dietary intake, or activity levels (*p* > 0.05). Sleep duration paralleled overall diet quality (*r* = 0.36, *p* = 0.04), whilst daytime sleepiness paralleled plasma lipids (*r* = 0.61, *p* =0.001) and sedentary time (*r* = 0.39, *p* = 0.02). Disturbances in sleep quality and plasma triglycerides were evident in non-obese asthmatic children referred for polysomnography, versus non-asthmatic children. Observed associations between diet quality, sedentary behavior, and metabolic and sleep-related outcomes warrant further investigation, particularly the long-term health implications.

## 1. Introduction

Overweight and obesity affects approximately 30% of children worldwide [1]. However, the prevalence of obesity in children with asthma has been reported at up to 45% [2]. Early intervention is essential to reduce the prevalence of overweight and obesity in children, particularly amongst the asthmatic population. 

The presence of asthma or wheeze may increase the risk of overweight and obesity in children and adolescents by 28%–40%, regardless of initial weight status, according to data from the Longitudinal Study of Australian Children [3]; yet, it remains unclear why children with asthma have a higher risk of obesity. Poor diet, short sleep duration, and high levels of sedentary behaviour can increase the risk for weight gain in children [4]. In addition, an inverse relationship between physical activity in preschool-aged children and incidence of overweight later in childhood has been highlighted in a systematic review of prospective studies [5]. Thus, modifiable lifestyle factors, including sleeping habits, dietary consumption patterns and activity behaviours, are a good target for weight gain prevention; however, whether lifestyle risk factors for weight gain are more prevalent in children with asthma has not been investigated.

Sleep disturbance has been recognised as a significant risk factor for weight gain in children [6] and, in non-asthmatic adults, has been linked to alterations in metabolic markers and appetite hormones [7], with an increased appetite for high-fat and high-sugar foods [7], increased daytime fatigue [8], and reduced physical activity [8] previously reported. Amongst children with asthma, one in five report problems of falling asleep, and one in ten report daytime sleepiness [9]. However, few studies have objectively assessed sleep disturbance in children using polysomnography [10], and, to our knowledge, the prevalence of modifiable weight-gain risk factors has not been compared between children, with and without asthma. 

This study aims to compare (i) sleep quantity and quality; (ii) metabolic biomarkers, food cravings, and dietary intake; and (iii) physical activity and sedentary behaviour, in children with and without asthma, referred for an overnight polysomnography. 

## 2. Materials and Methods 

### 2.1. Study Participants 

Non-obese children and adolescents (body mass index (BMI) *z*-score < 1.64) [11], aged 7–17 years, with (*n* = 17) and without (*n* = 17) asthma were recruited from the population referred to the Paediatric Sleep Unit (John Hunter Children’s Hospital, Newcastle NSW, Australia) for an overnight polysomnography. Asthma diagnosis was documented in the medical notes by the referring respiratory sleep physician. Exclusion criteria included medications known to interfere with sleep, e.g., antihistamines, methylphenidate; medical conditions, physical deformities, or genetic conditions that may interfere with sleep or negate participation in the study, e.g., cranio-facial abnormalities and Trisomy 21; diagnosed sleep disorders, e.g., narcolepsy, obstructive sleep apnoea; and current use of continuous positive airway pressure. Written informed participant assent and guardian consent were obtained. This study was approved by the Hunter New England and University of Newcastle Ethics Committees (10/08/18/5.04) and registered with the Australian and New Zealand Clinical Trials Registry (ACTRN12611000422921). 

### 2.2. Clinical Assessment

Subjects arrived at the Paediatric Sleep Unit for an overnight polysomnography between 16:00–18:00 h and departed between 06:00–07:30 h the following morning. Participants underwent clinical assessment as part of the study during this time, including medical history and medication use. Asthma stability was confirmed, defined as the absence of an exacerbation, respiratory tract infection, or use of oral corticosteroids in the preceding four weeks. Asthma control and severity were assessed using the Juniper Asthma Control Questionnaire [12] and Global Initiative for Asthma guidelines [13], respectively. Daytime sleepiness was assessed using the Paediatric Daytime Sleepiness Questionnaire [14]. Lung function was assessed using an EasyOne Spirometer (Model 2001 SN 67548/2008; Medizintechnik AG, Zurich, Switzerland) on the eve of the polysomnography and repeated the following morning. The greatest forced expiratory volume in 1 s (FEV_1_) and forced vital capacity (FVC) were selected from three technically acceptable manoeuvres according to American Thoracic Society guidelines and expressed as a percentage of predicted values [15]. 

### 2.3. Anthropometry 

Height was measured using a wall-suspended electronic stadiometer (Harpenden Stadiometers, Holtain Ltd., Crosswell, Pembrokeshire, UK). Weight was measured using a 200 kg max electronic, seated chair scale (Seca, Vogel & Hake, Hamburg, Germany). BMI was calculated (weight (kg)/height (m^2^)) and converted to BMI *z*-scores [11]. Waist circumference was measured to the nearest 0.1 cm at the midpoint between the lower costal edge and the iliac crest, using a non-extensible steel tape (Lufkin W606PM, Cooper Tools, Apex, NC, USA), and waist-to-height ratio was calculated (waist circumference (cm)/height (cm)). 

### 2.4. Polysomnography

Overnight polysomnography was performed using the modified 10–20 electroencephalogram application system and computerised sleep system (E-Series; Compumedics Ltd., Victoria, Australia) as previously described [16]. ‘Lights off’ was the participant’s usual bedtime and ‘lights on’ was 06:00 h. Polysomnography recordings were scored using Profusion Polysomnography 2 software (Compumedics Ltd., Victoria, Australia). Sleep-wake state and respiratory events were scored using the American Academy of Sleep Medicine Manual for the Scoring of Sleep and Associated Events 2007 [17]. Electroencephalogram arousals with a respiratory event (respiratory disturbance index; respiratory disturbance index in rapid eye movement (REM) sleep) and electroencephalogram arousals without a respiratory event (arousal index), were scored using modified American Thoracic Society criteria [18]. Use of these scoring guidelines was under the direction of the respiratory sleep physicians. Outcomes of interest were sleep quantity (total sleep time, total time awake), and sleep quality (% sleep efficiency, % REM sleep, sleep latency, arousal index, and respiratory disturbance index).

### 2.5. Dietary and Physical Activity Assessment

Food cravings were assessed using the Food Cravings Inventory-II [19], a 28-item questionnaire scored on a Likert scale of 1–5. The total score was obtained by averaging all 28 items, with a higher score indicating greater food cravings. Dietary intake was assessed using the validated food frequency questionnaire, the Australian Child & Adolescent Eating Questionnaire [20]. From this questionnaire, a diet quality score was derived, the Australian Recommended Food Score (Child and Adolescent) [21]. Time spent in physical activity per week (min) and average metabolic equivalent value were calculated using the Adolescent Physical Activity Recall Questionnaire [22]. Time spent in sedentary activity per week (min), e.g., passive travel, small-screen recreation and homework, was calculated using the Adolescent Sedentary Activity Questionnaire [23]. 

### 2.6. Metabolic Outcomes

A fasting blood sample was collected by a paediatric phlebotomist the morning following the polysomnography and centrifuged at 3000 rpm at 4 °C for 10 min. All samples underwent duplicate testing. Appetite hormones, plasma ghrelin, and leptin, were measured using commercial enzyme-linked immunosorbent assay (ELISA) (Bio-Rad, Hercules, CA, USA) with sensitivity of 100 pg/mL and 3.1 pg/mL, respectively. Inflammation was measured by serum high sensitivity C-Reactive Protein (hsCRP) (Dimension Vista System; Siemans Healthcare Diagnostics, Newark, NJ, USA). Plasma cortisol (Access Immunoassay Systems; Beckman Coulter, Fullerton, CA, USA), insulin growth factor-1 (Bioclone, Marrickville, NSW, Australia), insulin (Access Ultrasensitive Insulin assay; Beckman Coulter), and glucose (Dimension Vista System; Siemans Healthcare Diagnostics) were measured using their respective commercial assays, and homeostasis model assessment of insulin resistance (HOMA-IR) (glucose (mmol/L) × insulin (mlU/L)/22.5) calculated. Plasma cholesterol, high-density lipoprotein cholesterol (HDL), and triglycerides were also measured using commercial assays (Dimension Vista System; Siemans Healthcare Diagnostics).

### 2.7. Statistical Analysis

In order to detect a group difference of 90 pg/mL in plasma leptin with 80% power, we required *n* = 8 participants per group; however, accounting for a potentially low participation rate for blood collection and exclusion of participants post-polysomnography if a sleep disorder was diagnosed, we aimed to recruit *n* = 17 per group. Continuous data are presented as mean (standard deviation, SD) or median (interquartile range, IQR). Between-group comparisons were made using the Student’s *t*-test or Wilcoxon rank-sum test. Categorical data are presented as proportions (*n* (%)) and assessed using the Fischer’s exact test. Associations were assessed using Spearman’s rank correlation coefficients. Statistical significance was set at *p* < 0.05. Analysis was conducted using Intercooled Stata Version 11.0 for Windows (StataCorp, College Station, TX, USA). No adjustment for multiple testing was made. 

## 3. Results

Groups were similar in age, BMI *z*-score, and both morning and evening lung function (Table 1). However, there were more females in the non-asthmatic, compared to the asthmatic, group. Participants with asthma were well controlled, indicated by a median Asthma Control Questionnaire score of 0.6 (0.1, 1.0) units, and the majority classified with intermittent-mild asthma (86.7%). Maintenance treatment medication was used by 47.1% of children with asthma, with only 17.6% taking inhaled corticosteroids. Median inhaled steroid dose for the asthmatic group was 216.5 (200, 400) beclomethasone equivalents. Most children (64.7%) also reported use of a rescue short-acting beta-agonist. 

### 3.1. Sleep Outcomes

Sleep latency was significantly longer in the asthmatic group vs. the non-asthmatic group (Table 2), while total time awake, total sleep time, and sleep efficiency were similar between the groups. The Paediatric Daytime Sleepiness Scale score and the arousal indices (arousal index and respiratory disturbance index) were also similar between groups (Table 2). 

### 3.2. Metabolic Outcomes

**Figure 1:**
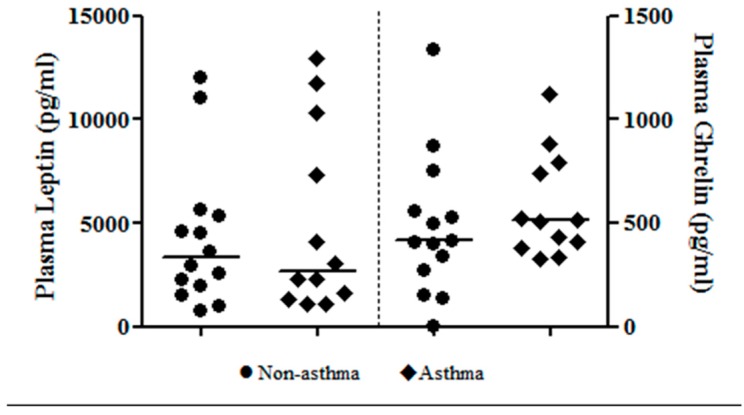
Fasting plasma levels of appetite hormones in children referred for an overnight polysomnography, asthma vs. non-asthma (ghrelin, *p* = 0.328; and leptin, *p* = 0.959).

Blood was collected in a subset of 13 asthmatic and 14 non-asthmatic children. Plasma triglycerides were significantly higher in the asthmatic group versus the non-asthmatic group (Table 3), while the HOMA-IR value and hsCRP were similar between groups. Morning plasma ghrelin (*p* = 0.328) and leptin (*p* = 0.959) levels were also similar between groups (Figure 1). 

### 3.3. Dietary Intake and Physical Activity

Food cravings, indicated by total Food Cravings Index-II score, did not differ between groups (2.1 (0.6) vs. 2.1 (0.8) units, *p* = 0.930). Dietary energy intake tended to be higher in the asthmatic group, but this was not statistically significant (Table 4). The proportion of energy consumed from macronutrients was similar between groups, as was the diet quality score (Australian Recommended Food Score) (Table 4). Although non-significant, the median weekly time spent in sedentary behaviour tended to be lower (1590 (1275, 3040) vs. 2160 (1755, 3000) min/week, *p* = 0.318) and physical activity tended to be higher (490 (263, 680) vs. 390 (210, 513) min/week, *p* = 0.174) in the asthmatic versus non-asthmatic group; while the average intensity of physical activity (4.5 (1.2) vs. 4.6 (1.5) metabolic equivalents, *p* = 0.701) was similar between groups. 

### 3.4. Correlations

Total sleep time was positively associated with diet quality score, while the Paediatric Daytime Sleepiness Scale score was positively associated with both the TC/HDL ratio and time spent in sedentary activity (Table 5). However, no associations between sleep variables and appetite hormones, food cravings score, time spent in physical activity, or the intensity of physical activity (metabolic equivalents) were detected. 

## 4. Discussion

The aim of this study was to explore whether the prevalence of lifestyle risk factors for weight gain are higher in children with asthma, compared to children without asthma. We examined several lifestyle risk factors, that is, sleep quantity and quality, appetite, food cravings and dietary intake, sedentary behaviour, and physical activity. In children referred for overnight polysomnography, sleep quality was impaired and plasma triglycerides increased in children with asthma, compared with non-asthmatic children; however, appetite hormones, dietary intake, and activity levels were similar between the two groups. Exploratory correlations suggest that sleep duration and daytime sleepiness may be associated with altered plasma lipids, dietary intake, and activity levels in children, with and without asthma.

Sleep quality was worse in children with asthma compared with non-asthmatic children, with a statistically significant difference in sleep latency of approximately 16 min. A similar difference has previously been reported between asthmatic and non-asthmatic children [16,24]; however, as the clinical importance of this difference remains unclear, future studies in a larger sample of children are warranted. Reduced sleep duration and increased nocturnal awakenings have been previously reported in children with asthma [25] and have indicated that both sleep disturbance and daytime sleepiness are associated with increasing asthma severity [26] and increased asthma symptoms [27]. Notably, children in the current study had well controlled, intermittent-mild severity asthma, which, in addition to the small sample size, may explain why a significant difference in sleep duration, sleep efficiency, arousals, or daytime sleepiness was not detected between the asthmatic and non-asthmatic groups.

Notably, fasting plasma triglycerides were 40% higher in children with asthma, compared to those without asthma, despite a similar dietary intake (specifically fat quantity and quality) and BMI *z*-score. Likewise, a previous study detected raised triglyceride levels in a greater proportion of asthmatic children compared with non-asthmatic children, and reported triglyceride levels to be associated with asthma prevalence, independent of BMI status [28]. An objective measure of body composition may have provided further insight to these findings, as higher adiposity has been reported in asthmatic children, compared with controls of a similar BMI *z*-score [29]. Although the triglyceride levels in the current study were not clinically abnormal, it does suggest that unfavourable changes in metabolic markers may occur in asthmatic children, despite a healthy weight status. Only 17.6% of the children in the current study were taking inhaled corticosteroids, and the indicator of insulin resistance (HOMA-IR) was similar between groups. Therefore, it is unlikely that the difference in triglycerides is due to inhaled corticosteroid use, impaired glucose tolerance, or insulin resistance. Moreover, the lack of correlation between plasma triglycerides and sleep quality and quantity suggests that altered sleep patterns may not be contributing to this metabolic impairment and further investigation is required. Indeed, this is an interesting observation as it may have important cardiovascular health implications in the future for this group of children.

The current study did not detect a significant difference between asthmatic and non-asthmatic children in plasma appetite hormones, food cravings, or time spent per week in physical activity or sedentary behaviour. However, in this group of children, both with and without asthma, daytime sleepiness paralleled the time spent in sedentary behaviour and the TC/HDL ratio, suggesting that increased sleepiness throughout the day is associated with increased sedentary behaviour and potentially adverse changes in plasma lipid levels. In contrast, there was no association between appetite markers and sleep variables. This is interesting given that previous studies have indicated that sleep disturbance may be associated with alterations in appetite [30,31]. In one study, food cravings measured by Food Craving Index-II were associated with increased daytime sleepiness in non-asthmatic adolescents [30]. A second study in non-asthmatic children found self-reported sleep duration correlated negatively with leptin concentrations in females, while no association was detected in males [31]. Unfortunately, our sample size was too small to investigate gender differences. Although our data indicated that a single night measure of sleep quantity and quality is not associated with appetite hormone concentrations or food cravings in this group of young children, future studies investigating the relationship between appetite markers and chronic sleep disturbance may be beneficial to this area of research. 

Supporting the hypothesis that sleep duration impacts diet quality, the current study found that a longer total sleep time was associated with a better diet quality score. This indicates that in those children with a greater sleep duration, dietary patterns are more closely aligned with the National Dietary Guidelines for Australian Children and Adolescents, reflecting a more optimum nutrient intake [21]. In agreement, recent studies in non-asthmatic children have indicated that increased sleep duration is associated with better dietary practices, including higher fruit and vegetable consumption and reduced soda consumption [32,33]. For example, one longitudinal study found that short sleep duration in children was associated with unfavourable dietary intake patterns in both girls and boys [33]. Notably, eating patterns were found to partially mediate the relationship between sleep and overweight/obesity in this group of children. 

The current study was a unique opportunity to access a paediatric population referred for objective sleep assessment and provides a preliminary exploration of lifestyle risk factors for weight gain in children with and without asthma, which has not been previously explored. A key strength of this study is the utilisation of a population undergoing polysomnography, which enabled an objective assessment of sleep quantity and quality, appetite, dietary intake, and activity measures in a clearly defined group of asthmatic and non-asthmatic children. Notably, this sample of children had suspected sleep disorders, which limits the external validity. However, the Paediatric Daytime Sleepiness Scale score and arousal indices were clinically normal, indicating that these children were without excessive daytime sleepiness or sleep disordered breathing. Furthermore, the sleep studies were reviewed by a specialist to confirm no clinically abnormal alternations were present, nor diagnosis of sleep disorder given. Limitations in the current study include the small sample size, the higher number of females in the non-asthmatic group, and the potential ‘first-night effect’ of the sleep lab environment. Furthermore, this study is cross-sectional in nature and is unable to establish the direction of associations or indicate the effects of chronic sleep disturbance. Chronic sleep disturbance may be more closely associated with changes in lifestyle patterns, and further research is required. 

This work provides a preliminary look at risk factors for weight gain in children with asthma, which has not been previously described. Sleep quality, but not sleep quantity, was poorer in children with asthma, compared to children without asthma. In addition, triglyceride levels were marginally elevated in asthmatic compared with non-asthmatic children, despite similar dietary intakes and BMI *z*-score, and this aspect requires further investigation. However, there were no differences in appetite markers, dietary intake, or activity levels between children with and without asthma. Daytime sleepiness correlated with elevated lipid levels and increased sedentary activity, which, if chronically sustained, could lead to a positive energy balance. This warrants further investigation, particularly in groups with excessive daytime sleepiness. Sleep duration was also correlated with dietary consumption patterns in accordance with national dietary guidelines. However, further investigation into sleep disturbance in asthmatic children and the lifestyle implications, with the consideration of gender, is warranted. For researchers and clinicians, it is important to continue the investigation into the aetiology of the increased prevalence of overweight and obesity in children with asthma, in order to prevent further complications to the clinical management of this group of children.

## Figures and Tables

**Table 1:** Subject characteristics and lung function of children with and without asthma, referred for an overnight polysomnography.

Subject Characteristics	Non-Asthma (*n* = 17)	Asthma (*n* = 17)	*p* Value
Age (years); mean (SD)	10.8 (2.3)	10.7 (2.4)	0.928
Gender (female); *n* (%)	12 (70.6)	5 (29.4)	0.016 ^1^
Height (cm); median (IQR)	146.0 (136.7, 153.0)	139.8 (133.0, 147.7)	0.380
Weight (kg); median (IQR)	37.0 (30.5, 46.1)	36.0 (28.1, 41.6)	0.502
BMI *z*-score (SD); median (IQR)	0.4 (−1.2, 1.0)	0.0 (−0.4, 0.6)	0.986
Waist-to-height (cm/cm); mean (SD)	0.44 (0.06)	0.46 (0.05)	0.228
*Evening lung function*			
FEV_1_ %predicted (%); mean (SD)	94.0 (12.7)	93.8 (17.3)	0.974
FVC %predicted (%); mean (SD)	90.8 (11.7)	93.3 (11.9)	0.552
FEV_1_/FVC (%); mean (SD)	87.2 (86.4, 91.5)	86.0 (77.8, 91.1)	0.207
*Morning lung function*			
FEV_1_ %predicted (%); mean (SD)	93.8 (12.3)	88.1 (14.0)	0.258
FVC %predicted (%); mean (SD)	90.7 (7.9)	90.3 (10.0)	0.914
FEV_1_/FVC (%); mean (SD)	88.0 (84.5, 89.8)	79.2 (75.7, 88.7)	0.125

^1^ Significantly different between asthma and non-asthma group (*p* < 0.05); BMI: body mass index; FEV_1_: forced expiratory volume in 1 s; FVC: forced vital capacity; SD: standard deviation; IQR: interquartile range.

**Table 2:** Sleep quantity and quality in children with and without asthma, referred for an overnight polysomnography.

Sleep Indices	Non-Asthma (*n* = 17)	Asthma (*n* = 17)	*p* Value
Paediatric Daytime Sleepiness Scale (score); mean (SD)	15.3 (5.2)	16.2 (5.7)	0.620
Total sleep time (min); mean (SD)	428.5 (38.8)	415.3 (53.5)	0.416
Total awake time (min); median (IQR)	44.0 (35.0, 54.5)	54.5 (32.0, 66.5)	0.642
Sleep efficiency (%); mean (SD)	81.6 (6.9)	78.2 (7.5)	0.181
Sleep latency (min); mean (SD)	40.9 (16.9)	56.6 (25.5)	0.042 ^1^
*Non-REM Sleep Stages:*			
Stage 1 (%); median (IQR)	2.0 (0.7, 2.6)	0.6 (0.3, 1.6))	0.094
Stage 2 (%); median (IQR)	48.9 (43.3, 52.6)	49.0 (43.5, 55.8)	0.931
Stage 3 (%); median (IQR)	31.4 (29.0, 37.1)	32.7 (26.1, 38.1)	0.904
REM latency (min); median (IQR)	166.5 (139.0, 237.5)	149.0 (100.5, 192.0)	0.294
REM sleep (%); mean (SD)	17.9 (8.5)	16.9 (6.4)	0.717
Arousal Index (*n*/h); median (IQR)	3.2 (1.9, 10.1)	3.7 (2.7, 6.4)	0.836
Respiratory Disturbance Index (*n*/h); median (IQR)	0.1 (0.0, 1.3)	0.2 (0.0, 0.4)	0.986

^1^ Significantly different between asthma and non-asthma group (*p* < 0.05); REM: rapid eye movement.

**Table 3:** Metabolic biomarkers in children with and without asthma, referred for an overnight polysomnography.

Systemic Biomarkers	Non-Asthma (*n* = 14)	Asthma (*n* = 13)	*p* Value
Cortisol (nmol/L); median (IQR)	363 (310, 424)	326 (310, 403)	0.865
IGF-1 (U/ml); median (IQR)	1.04 (0.74, 1.94)	0.73 (0.66, 1.08)	0.190
hsCRP (mg/L); median (IQR)	1.1 (0.5, 1.5)	0.7 (0.2, 0.9)	0.113
HOMA-IR; median (IQR)	1.4 (0.9, 2.4)	1.4 (1.0, 1.7)	0.961
Triglycerides (mmol/L); median (IQR)	0.7 (0.7, 0.8)	1.0 (0.8, 1.2)	0.013 ^1^
Cholesterol (mmol/L); mean (SD)	4.4 (0.4)	4.8 (0.2)	0.380
TC/HDL (ratio); mean (SD)	3.2 (1.0)	3.3 (0.9)	0.786

^1^ Significantly different between asthma and non-asthma group (*p* < 0.05); hsCRP: high-sensitivity C-Reactive Protein; HOMA-IR: homeostatic model assessment of insulin resistance; IGF: insulin growth factor; TC/HDL: total cholesterol/high-density lipoprotein cholesterol

**Table 4:** Dietary intake measured by the Australian Child and Adolescent Eating survey in children with and without asthma, referred for an overnight polysomnography.

Dietary Intake Measures	Non-Asthma (*n* = 17)	Asthma (*n* = 17)	*p* Value
Energy (kj); mean (SD)	8945.5 (2314.5)	9365.4 (2722.7)	0.631
Protein (%energy); mean (SD)	16.4 (2.5)	17.2 (2.5)	0.380
Total fat (%energy); mean (SD)	33.4 (4.1)	31.5 (3.7)	0.183
Saturated fat (%fat); mean (SD)	51.0 (3.5)	49.1 (4.4)	0.177
PUFA (%fat); mean (SD)	11.7 (2.1)	12.7 (3.1)	0.276
MUFA (%fat); mean (SD)	37.2 (2.3)	38.2 (2.2)	0.197
Carbohydrate (%energy); mean (SD)	51.6 (5.3)	52.0 (4.5)	0.809
Sugars (g); mean (SD)	146.4 (47.7)	159.4 (72.6)	0.542
Fibre (g); mean (SD)	23.5 (6.4)	27.2 (8.4)	0.151
Australian Recommended Food Score; mean (SD)	26.6 (8.1)	27.9 (10.1)	0.683

MUFA: mono-unsaturated fatty acid; PUFA: poly-unsaturated fatty acid.

**Table 5:** Correlations between key sleep variables and metabolic, dietary, and physical activity markers in children with and without asthma.

Correlations	Total Sleep Time (min)	Paediatric Daytime Sleepiness Scale (Score)
Ghrelin (pg/mL)	0.215	−0.0003
Leptin (pg/mL)	−0.377	0.143
TC/HDL (ratio)	−0.090	0.614 ^1^
Triglycerides (mmol/L)	−0.159	0.307
Food Cravings Index-II (score)	0.106	−0.042
Australian Recommended Food Score	0.357 ^1^	−0.016
Physical activity (min/week)	0.315	−0.143
Physical activity intensity (METS)	0.107	0.077
Sedentary activity (min/week)	−0.270	0.386 ^1^

Spearman rank correlation coefficients, *r*, are presented. ^1^ Indicates a *p* value < 0.05; TC/HDL: total cholesterol/high-density lipoprotein cholesterol; METS: metabolic equivalents.
